# THE RELATIONSHIP OF COGNITIVE CONTROL BELIEFS TO 10 YEAR CHANGES IN COGNITIVE PERFORMANCE

**DOI:** 10.1093/geroni/igad104.2631

**Published:** 2023-12-21

**Authors:** Kylie Schiloski, Margie Lachman

**Affiliations:** Brandeis University, Waltham, Massachusetts, United States; Brandeis University, Waltham, Massachusetts, United States

## Abstract

Past work has shown that older adults who believe they have control over their cognitive performance (i.e., higher cognitive control beliefs) have better cognitive performance than those with lower cognitive control beliefs. However, it is unclear whether cognitive control beliefs are related to individual differences in cognitive change among middle-aged and older adults. The current study examined longitudinal associations between cognitive control beliefs and 10-year cognitive changes using data from waves 2 and 3 of the Midlife in the United States study (MIDUS; N=2,509, Ages 33 to 83 at wave 2). We predicted that higher baseline cognitive control beliefs would be related to greater maintenance of episodic memory and executive functioning over 10 years. Age was tested as a moderator of these relationships and each analysis controlled for baseline performance, age, sex, education, and self-rated physical health. Multiple linear regression models revealed that higher baseline control beliefs were related to less decline in episodic memory (p<.001) and executive functioning (p<.05); however, age did not moderate these relationships. Taken together, these results suggest that across age, cognitive control beliefs are associated with greater maintenance of episodic memory and executive functioning over 10 years in middle-aged and older adults. As such, cognitive control beliefs may represent a promising modifiable mechanism to help minimize cognitive declines starting in midlife.

